# Genetic predisposition to type 2 diabetes is associated with severity of coronary artery disease in patients with acute coronary syndromes

**DOI:** 10.1186/s12933-019-0930-1

**Published:** 2019-10-08

**Authors:** Qiwen Zheng, Jie Jiang, Yong Huo, Dafang Chen

**Affiliations:** 1 Department of Epidemiology and Biostatistics, School of Public Health, Peking University, No. 38 Xueyuan Road, Haidian District, Beijing, 100191 China; 2 Department of Cardiology, Peking University First Hospital, No. 8 Xishiku Street, Xicheng District, Beijing, 100034 China

**Keywords:** Acute coronary syndromes, Atherosclerosis, Coronary artery disease, Genetic risk score, Severity, Type 2 diabetes

## Abstract

**Background:**

Accumulating evidence has shown that type 2 diabetes (T2D) and coronary artery disease (CAD) may stem from a ‘common soil’. The aim of our study was to examine the association between genetic predisposition to T2D and the risk of severe CAD among patients with acute coronary syndromes (ACS) undergoing angiography.

**Methods:**

The current case–control study included 1414 ACS patients with at least one major epicardial vessel stenosis > 50% enrolled in the ACS Genetic Study. The severity of CAD was quantified by the number of coronary arteries involved. Genetic risk score (GRS) was calculated using 41 common variants that robustly associated with increased risk of T2D in East Asians. Logistic regression models were used to estimate the association between GRS and the severity of CAD.

**Results:**

In the age-, sex- and BMI-adjusted model, each additional risk allele was associated with a 6% increased risk of multi-vessel disease (OR = 1.06, 95% CI 1.02–1.09). The OR was 1.43 (95% CI 1.08–1.89) for the risk of severe CAD when comparing the extreme tertiles of T2D-GRS. The association was not reduced after further adjustment for conventional cardiovascular risk factors. Additional adjustment for T2D status in our regression model attenuated the association by approximately one quarter. In subgroup analysis, the strengths of the associations between GRS and the severity of CAD were broadly similar in terms of baseline demographic information and disease characteristics.

**Conclusions:**

Our data indicated that genetic predisposition to T2D is associated with elevated risk of severe CAD. This association revealed a possible causal relationship and is partially mediated through diabetic status.

## Background

The increasing prevalence of type 2 diabetes (T2D) and coronary artery disease (CAD) has become a global public health concern. T2D and CAD are now leading causes of mortality and morbidity worldwide [1, 2]. Accumulating evidence has shown that T2D and CAD might stem from a ‘common soil’, where both conditions shared a common genetic background and lifestyle risk factors (e.g. obesity, high blood pressure, cigarette smoking, and a lack of physical exercise) [3]. Population-based observational studies have confirmed that patients with T2D have an increased risk of developing cardiovascular disease (CVD) and cardiac death compared with non-diabetic people [4–7]. Furthermore, previous studies have revealed a significant association between T2D-predisposing variants and increased risk of CAD in the general population [8] and in people with T2D [9]. Recent Mendelian randomization analyses further support a causal role for T2D on CAD [10, 11].

Recently genome-wide association studies (GWAS) have identified numerous genetic variants associated with T2D [12–15]. Considering a single variant confers modest risk, combining multiple variants as a genetic risk score (GRS) is useful for predicting incidence of T2D [16, 17], future CAD [8], and all-cause mortality [18]. Observational studies have demonstrated that diabetes is associated with a higher prevalence CAD. Moreover, such studies have also shown that diabetes is linked with the severity of CAD [19–21], which is highly associated with increasing mortality and morbidity in long-term outcomes [22]. However, it remains unclear whether T2D plays a causal role in severe CAD. Therefore, utilization of GRS to assess the association between T2D and severe CAD might clarify the causality, improve risk reclassification, and shed light on future therapeutic direction.

Our aim in this study was to construct a GRS based on multiple variants predisposing to T2D and examine the association between GRS and the severity of CAD in patients with acute coronary syndromes (ACS) from a Chinese prospective cohort: the Acute Coronary Syndrome Genetic study (ACS Genetic Study). We further investigated whether this association was modified by baseline characteristics, including demographic information, lifestyle factors, and comorbidities.

## Methods

### Study participants

The ACS Genetic study is a prospective, observational cohort study that consisting of 1803 ACS patients who were recruited at hospital discharge during January 2011 to December 2013. Details of this study (NCT01964313) have been described elsewhere [23]. Briefly, eligible patients were adults (18 years or older) who were hospitalized within 48 h of symptom onset of the index event. In addition, discharge diagnosis of ST-elevation myocardial infarction (STEMI), non-ST-elevation myocardial infarction (NSTEMI), or unstable angina (UA) were required to meet prespecified criteria (Additional file 1: Table S1). Patients were excluded if their ACS events were the result of a complication or if it occurred during hospitalization due to other reasons. Other exclusion criteria were any impeding causes of follow-up, any serious comorbidities limiting life expectancy to less than 6 months, or enrolment in another clinical trial. Baseline information regarding demographics, medical history, utilization of medications, event characteristics, and treatment procedures were collected by the investigators and documented on the electronic case report forms. Information about health and disease after discharge were collected via telephone interviews every 3-month until up to 5 years after the ACS index event. All participants provided written informed consent in accordance with the Declaration of Helsinki. The study protocol was approved by the Medical Ethics Committee of Peking University First Hospital.

For the purpose of this study, only patients who underwent coronary angiography with at least one epicardial stenosis > 50% were eligible. Therefore, participants who did not have coronary angiography (n = 224) or significant angiographic CAD (stenosis < 50% in any vessels, n = 29) were removed from further analysis.

### Outcome and covariate definitions

Coronary angiography was performed using standard procedures and techniques. The coronary angiograms were reviewed by two independent angiographers who were both blinded to the results of the genotype. Significant coronary artery stenosis was defined as at least 50% stenosis in any of the major vessels (left main coronary artery, left anterior descending artery, left circumflex artery, or right coronary artery). The primary outcome of the present study was the severity of CAD. The severity of CAD in each subject was quantified by the number of coronary arteries involved. Single-vessel disease was defined as at least 50% stenosis in one major coronary artery. Multi-vessel disease was defined as at least 50% stenosis in two or more major coronary arteries. A 50% or more reduction in the internal diameter of the left main coronary artery was considered to be three-vessel disease.

T2D cases in our study were defined as self-report of doctor-diagnosed T2D and/or antidiabetic medication use. Similarly, patients were defined as having hypertension or hypercholesterolemia if they self-reported doctor-diagnosed hypertension or hypercholesterolemia or were prescribed antihypertensive therapy or lipid lowering therapy, respectively. Current smokers were defined as patients who continued to smoke (> 1 cigarette per day) at the time of enrolment or quit within the past 30 days. Body mass index (BMI) was calculated as weight divided by the squared height measurement at the time of hospital admission. Family history of CAD was self-reported and defined as having any first-degree relatives (parents or siblings) with history of CAD at any age. Other comorbidities and medical histories, such as heart failure, chronic angina, and chronic renal failure, were defined based on self-reporting and/or detailed chart review by investigators.

### Genotyping and imputation

Single nucleotide polymorphisms (SNPs) genotyping has been described in detail elsewhere. In brief, samples were genotyped on the Infinium HumanExome BeadChip v1.2 (Illumina, San Diego, CA). Genotypes were called using the GenTrain version 2.0 of GenomeStudio software (version 2011.1; Illumina). Individuals with low genotyping call rate (< 95%), biological relatedness, duplication, gender mismatch, possible contamination or departure from Chinese Han population were removed before further analysis (n = 136). SNPs were excluded if they were monomorphic, duplicate, not on autosomal chromosomes, had a missing call rate ≥ 5%, or had a Hardy–Weinberg equilibrium (HWE) *P* value less than 1 × 10^−5^. Imputation of unmeasured SNPs were performed using IMPUTE2 with the recently released 1000 Genomes Project phase 3 (http://mathgen.stats.ox.ac.uk/impute/1000GP_Phase3/) as a reference panel [24, 25]. SNPs with low imputation quality scores (info < 0.7) or departure from HWE were further excluded.

### SNP selection and genetic risk score construction

Considering the heterogeneity in the linkage disequilibrium structure and minor allele frequency among different ethnicities, we selected the SNPs that have been identified in Europeans and successfully replicated in East Asians [26–28] or those that were identified and validated in meta-analyses including GWASs from East Asians [13, 29]. These include, PROX1 rs340874, NOTCH2 rs10923931, GRB14 rs13389219, BCL11 rs243021, IRS1 rs2943641, GCKR rs780094, THADA rs7578597, PPARG rs1801282, IGF2BP2 rs4402960, ADCY5 rs11708067, MAEA rs6815464, ANKRD55 rs459193, CDKAL1 rs9356744, KCNK16 rs1535500, JAZF1 rs864745, DGKB rs2191349, PAX4 rs10229583, SLC30A8 rs13266634, TP53INP1 rs896854, ANK1 rs515071, CDKN2A/B rs10811661, PTPRD rs17584499, GLIS3 rs7041847, TLE1 rs2796441, TCF7L2 rs7903146, ZMIZ1 rs12571751, HHEX/IDE rs1111875, CDC123/CAMK1D rs10906115, KCNQ1 rs2237892, ARAP1 rs1552224, KCNJ11 rs5215, KLHDC5 rs10842994, SPRY2 rs1359790, C2CD4A/C2CD4B rs7172432, HMG20A rs7178572, PRC1 rs8042680, FTO rs17817449, TCF2 (HNF1B) rs4430796, SRR rs391300, SLC16A11 rs13342232, MC4R rs12970134, and PEPD rs3786897 (Additional file 1: Table S2). These SNPs all reached a genome-wide significance level, and no linkage disequilibrium relationship existed among the above loci. We tested the HWE as described by Nielsen et al. [30]. The genotype distributions of the above SNPs did not deviate significantly from the HWE (P ≥ 0.001 = 0.05/42, Additional file 1: Table S2) except for rs7041847 at GLIS3. Therefore, we used 41 T2D associated SNPs to construct the GRS. The characteristics of the individual SNP and the association of each SNP with T2D are shown in Additional file 1: Table S2. We assumed that each SNP in the panel acts independently in an additive manner, and the GRS was calculated using a weighted method. Each SNP was weighted according to its relative effect size (β coefficient) obtained from the reported meta-analysis data [13]. Weighted score was created by multiplying each β coefficient by the number of corresponding risk alleles and then summing the products. In order to make the GRS easier to interpret, we rescaled the weighted score to reflect the number of risk alleles. Therefore, each point of the GRS corresponded to one risk allele and individual with a higher GRS represented higher predisposing genetic risk to T2D. We also constructed an unweighted GRS by summing up the number of risk alleles for the sensitivity analysis only.

### Statistical analysis

Medians and interquartile ranges were reported for continuous variables. Frequencies and proportions were reported for categorical variables. We used t tests (or Mann–Whitney U tests with non-normally distributed data) to compare continuous data, and χ^2^ tests to compare categorical demographic and clinical variables in patients with multi-vessel disease and controls. Multivariable logistic regression models were applied to examine the association between GRS and the risk of CAD severity. Model 1 was adjusted for age, sex, and BMI. Model 2 was further adjusted for current smoker, hypertension, and hypercholesterolemia. Model 3 was additionally adjusted for the status of T2D in order to examine whether the causal relationship between T2D and severity of CAD existed, because attenuation of the associations between GRS and severity of CAD after adjustment for T2D would be suggestive of causality between T2D and risk of multi-vessel disease. To further examine whether the observed effect of GRS on the severity of CAD was in agreement with the expected effect based on the effect size of GRS on T2D and the effect size of T2D on severity of CAD, we calculated the expected effect size according to the following equation: β_E_ = β_G_ × β_D_. Where β_E_ is the expected effect size of GRS on the severity of CAD, β_G_ is the effect size of GRS on T2D, and β_D_ is the effect size of T2D on multi-vessel disease risk. Student’s t test was used to compare the difference of expected effect size and the observed effect size [31–34]. Multinomial logistic regression models were fitted to assess the associations of the GRS with the number of vessels involved (one-vessel, two-vessel and three-vessel). To examine the accumulative effect of the GRS, we compared the multi-vessel disease risk across the tertile of the GRS. Linear relation analysis between the GRS (as a continuous variable) and the risk of multi-vessel disease was performed by using a restricted cubic spline regression model [35]. To investigate whether the association of GRS with the severity of CAD differs in participants with various baseline characteristics, including demographic information, lifestyle factors, and comorbidities, we assessed the interaction of GRS and aforementioned characteristics by adding an interaction term in the regression model. The interaction term was the product of the interaction factor and the GRS. Moreover, we performed a series of stratified analyses by separately studying the association of the GRS with the severity of CAD in patients with and without prespecified characteristics. We investigated whether the individual SNP has a potential pleiotropic effect by searching the NHGRI-EBI GWAS Catalog. In the sensitivity analysis, we investigated the association between GRS and severity of CAD by using the unweighted GRS and the GRS excluding the SNPs that were associated with other metabolic traits (including BMI, WHR, CAD, TC, TG, HDL, LDL) reported by NHGRI-EBI GWAS Catalog. Missing covariates were multiply imputed using Multiple Imputation by Chained Equations (MICE) [36]. Nine imputed sets were generated using all baseline characteristics in the models. We used Quanto for power calculations [37]. The study had 80% power to detect the per-allele effect of T2D-GRS on severe CAD with the corresponding odds ratio (OR) of 1.04 at a significance level of 0.05. All analyses were carried out with R statistical software version 3.5.0 (http://www.r-project.org). The reported significance levels were all two-sided, with statistical significance set at 0.05.

## Results

### Characteristics of the study participants

Table 1 shows the characteristics of the study participants. Of the 1414 ACS patients, 803 (56.8%) had UA, 340 (24.0%) had STEMI, and 271 (19.2%) had NSTEMI. Consistent with previous studies, patients with multi-vessel disease were more likely to be elderly, have a heavy burden of traditional risk factors (i.e. hypertension, diabetes, hypercholesterolemia or cigarette smoking), and have prior history of vascular disease.

**Table 1:** Baseline characteristics among 1414 patients with ACS

Variables	Total	Single-vessel disease	Multi-vessel disease	*P* value
No. of patients	n = 1414	n = 490	n = 924	
Demographics				
Age, median (IQR), years	60 (53–68)	58 (49–65)	61 (55–69)	< 0.001
BMI, median (IQR), kg/m^2^	24.7 (22.7–26.9)	24.7 (22.5–26.8)	24.7 (22.9–27.0)	0.325
Male, n (%)	1112 (78.6)	380 (77.5)	732 (79.2)	0.509
Current smoking, n (%)	588 (41.6)	201 (41.0)	387 (41.9)	0.798
Diagnosis, n (%)				
NSTEMI	271 (19.2)	89 (18.2)	182 (19.7)	
STEMI	340 (24.0)	113 (23.1)	227 (24.6)	0.544
UA	803 (56.8)	288 (58.8)	515 (55.7)	
Medical history, n (%)				
Hypertension	753 (53.3)	219 (44.7)	534 (57.8)	< 0.001
Type 2 diabetes	288 (20.4)	69 (14.1)	219 (23.7)	< 0.001
Hypercholesterolemia	255 (18.0)	81 (16.5)	174 (18.8)	0.318
Prior myocardial infarction	121 (8.6)	34 (7.0)	87 (9.4)	0.138
Prior revascularization	112 (7.9)	32 (6.5)	80 (8.6)	0.192
Stroke/transient ischemic arrack (TIA)	71 (5.0)	19 (3.9)	52 (5.6)	0.192
Heart failure	12 (0.8)	2 (0.4)	10 (1.1)	0.236
Chronic angina	168 (11.9)	43 (8.8)	125 (13.5)	0.011
Chronic renal failure	8 (0.6)	3 (0.6)	5 (0.5)	1.000
Chronic obstructive pulmonary diseases (COPD)/other chronic lung diseases (CLD)	8 (0.6)	4 (0.8)	4 (0.4)	0.459
Peripheral vascular disease	11 (0.8)	2 (0.4)	9 (1.0)	0.348
Genetic information				
Family history of CAD, n (%)	117 (8.3)	36 (7.3)	81 (8.8)	0.412
T2D-GRS, median (IQR)	48.5 (46.0–51.1)	48.1 (45.5–50.5)	48.7 (46.3–51.3)	< 0.001

The T2D-GRS ranged from 35.5 to 61.0, and the median (interquartile range) was 48.5 (46.0–51.1) in all subjects. As expected, higher GRS were associated with higher OR of T2D risk and higher fasting glucose (Additional file 1: Figures S1 and S2). After adjustment for age, sex and BMI, the GRS was significantly associated with T2D. The OR associated with each additional point of score was 1.09 (95% CI 1.05–1.13, *P *< 0.001).

### GRS, T2D and severity of CAD risk

In the age, sex, and BMI adjusted model, T2D was significantly associated with an increased risk of multi-vessel disease. After adjustment for potential confounders including smoking status, hypertension, and hypercholesterolemia, the associations were slightly attenuated but still persisted. Compared with participants without T2D, in individuals with T2D the multivariate-adjusted OR was 1.69 (95% CI 1.25–2.32, *P* < 0.001) for developing multi-vessel disease (Table 2).

**Table 2:** The associations of present T2D and T2D-GRS with multi-vessel disease

	T2D cases/total subjects	Model 1^a^		Model 2^b^		Model 3^c^	
		OR (95% CI)	*P*	OR (95% CI)	*P*	OR (95% CI)	*P*
Present T2D	288/1414	1.79 (1.33–2.44)	< 0.001	1.69 (1.25–2.32)	< 0.001	–	–
Continuous variable of GRS							
Per SD (3.81 points)		1.23 (1.10–1.37)	< 0.001	1.21 (1.08–1.36)	< 0.001	1.16 (1.03–1.31)	0.012
Per risk allele		1.06 (1.02–1.09)	< 0.001	1.05 (1.02–1.08)	< 0.001	1.04 (1.01–1.07)	0.012
Categorical variable of GRS							
Low risk	43/472	1.00	0.011	1.00	0.021	1.00	0.126
Medium risk	78/471	1.12 (0.86–1.47)		1.11 (0.84–1.46)		1.08 (0.82–1.42)	
High risk	167/471	1.43 (1.08–1.89)		1.39 (1.05–1.84)		1.25 (0.94–1.67)	

^a^Adjust for age, sex and BMI

^b^Adjust for age, sex, BMI, smoking, hypertension, and hypercholesterolemia

^c^Adjust for age, sex, BMI, smoking, hypertension, hypercholesterolemia, and T2D

**Fig. 1:**
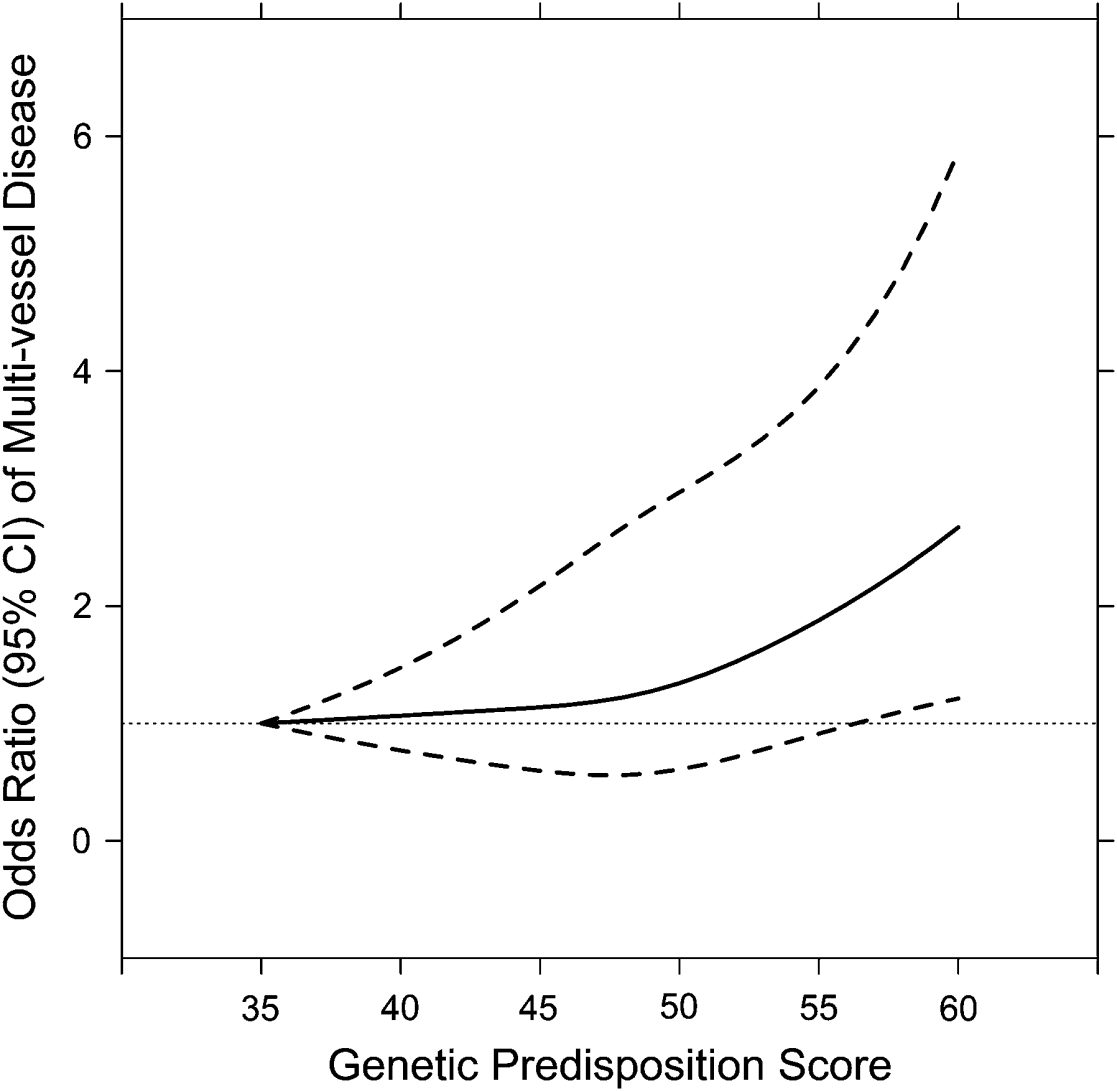
Linear relationship between T2D-GRS and risk of multi-vessel disease. Data are OR (solid lines) and 95% CI (dashed lines), adjusted for age, sex, BMI, current smoker, hypertension, and hypercholesterolemia

**Fig. 2:**
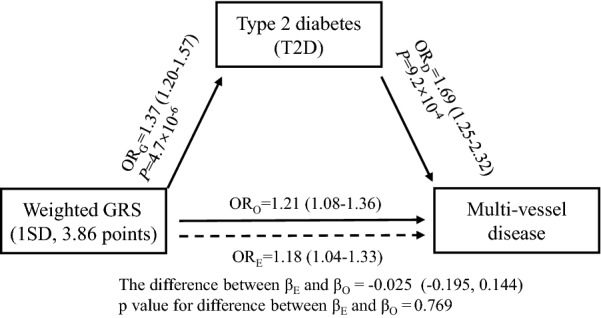
Triangular relationship among GRS, T2D and multi-vessel disease. OR_G_ represented the association between GRS and T2D risk after adjustment for age, sex, and BMI; OR_D_ represented the association between T2D and multi-vessel disease; OR_O_ represented the observed association of GRS with multi-vessel disease; and OR_E_ represented the expected association of GRS with multi-vessel disease. OR_D_, OR_O_, and OR_E_ were calculated after adjustment for age, sex, BMI, smoking status, hypertension, and hypercholesterolemia

The associations of each individual SNP with risk of multi-vessel disease are reported in Additional file 1: Figure S3. Most of the individual SNPs did not show significant association with severity of CAD risk, except for three loci (PROX1, NOTCH2, and HMG20A). In order to overcome the limitation of lacking power in single risk allele approach, we used the GRS to explore the aggregate effect of genetic predisposition to T2D on multi-vessel disease risk. As shown in Table 2, each standard deviation (SD, 3.81 points) increase in GRS was associated with a 23% increased risk of multi-vessel disease in age, sex, and BMI adjusted model (95% CI 1.10–1.37, *P* < 0.001, model 1). Further adjustment for smoking status, hypertension, and hypercholesterolemia did not substantially change the results (OR 1.21, 95% CI 1.08–1.36, *P* < 0.001, model 2). In addition, we also used a restricted cubic spline regression model to explore the association continuously. The results showed a linear relationship between the GRS and increased risk of multi-vessel disease in ACS patients (Fig. 1, *P* for nonlinearity is 0.350). The ORs for the severity of CAD risk significantly increased across the tertile of the GRS (*P* for trend = 0.021). Compared to the participants in the low-risk group, subjects in the high-risk group had an OR of 1.39 (95% CI 1.05–1.84), adjusted for age, sex, BMI and other traditional risk factors. When classifying multi-vessel disease as one-, two- and three-vessel disease, we found a stronger association between a high GRS and 3-vessel disease than with 2-vessel disease (Table 3). The association between GRS and multi-vessel disease was attenuated by approximately one quarter (OR 1.16, 95% CI 1.03–1.31) when prevalent T2D was also included as a covariate in the regression model (model 3). The association between GRS and multi-vessel disease was not different from the association expected based on the observed association between GRS and T2D, and the observed association between T2D and multi-vessel disease (*P* = 0.769). The triangular relationship between the T2D, GRS and multi-vessel disease indicated that this association might be partially mediated through T2D (Fig. 2).

**Table 3:** The association of GRS (per SD, 3.81 points) with number of vessels involved

Model	Number of vessels involved		
	1	2	3
Model 1^a^			
OR (95% CI)	1.00	1.17 (1.02–1.34)*	1.27 (1.12–1.45)***
Model 2^b^			
OR (95% CI)	1.00	1.17 (1.02–1.34)*	1.25 (1.10–1.43)***
Model 3^c^			
OR (95% CI)	1.00	1.10 (0.96–1.27)	1.22 (1.06–1.39)**

* *P* < 0.05; ** *P* < 0.01; *** *P* < 0.001

^a^Adjust for age, sex and BMI

^b^Adjust for age, sex, BMI, smoking, hypertension, and hypercholesterolemia

^c^Adjust for age, sex, BMI, smoking, hypertension, hypercholesterolemia, and T2D

### Stratified analysis

**Fig. 3:**
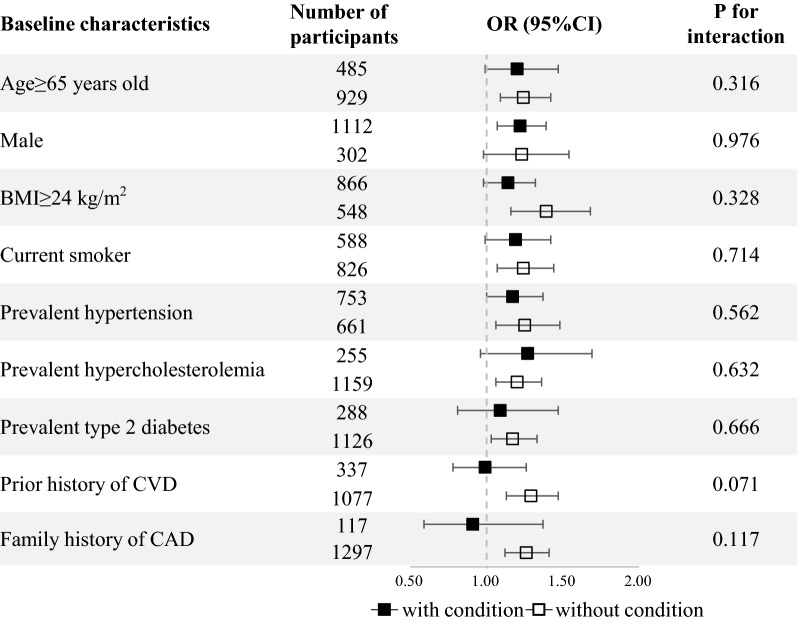
Association between GRS and severe CAD in subgroups of the participants with and without demographic characteristics, lifestyle factors, and comorbidities. All ORs were calculated per SD increase in GRS. All analyses were adjusted for age and sex, except for age and sex subgroup. Prior CVD included prior myocardial infarction, prior revascularization, heart failure, stroke/transient ischemic arrack, chronic angina and peripheral vascular disease

We next investigated whether the association between genetic predisposition to T2D and severity of CAD risk varied across subgroups of the participants stratified by demographic characteristics, lifestyle factors, and comorbidities (Fig. 3). There was no statistical difference in the strength of the association between GRS and multi-vessel disease in the subgroups of participants stratified by age, gender, BMI, smoking status, prevalent diseases, and family history of CAD (all *P* for interaction > 0.1). We found that the association was more pronounced in participants with absent disease history of CVD though there was no significant interaction (*P* for interaction = 0.071).

### Sensitivity analyses

To further investigate the association between GRS and severity of CAD, we undertook several sensitivity analyses. We observed a consistent and significant association between genetically determined T2D and increased risk of multi-vessel disease with an OR of 1.21 (95% CI 1.08–1.36, *P* < 0.001) using the unweighted GRS (Additional file 1: Table S3). Additionally, in order to explore whether the association between GRS and the severity of CAD was influenced by the genes that have been shown to be linked to CAD or other risk factors, we further performed an additional analysis by excluding 13 pleiotropic SNPs (Additional file 1: Table S2). The GRS_no pleiotropic SNP_ was associated with severity of CAD in both continuous and categorical variable analysis after adjustment for age, sex, BMI, smoking, hypertension, and hypercholesterolemia (Additional file 1: Table S3).

## Discussion

In this cross-sectional study, we examined the association between a genetic score comprising 41 independent T2D risk variants and severity of CAD. We found that genetic predisposition to T2D significantly increased the risk of severe CAD, independent of disease status and lifestyle risk factors for CAD. Our results revealed the possible causal relationship between T2D and severe CAD.

### The association between GRS and severe CAD

In our study, we observed each additional T2D-increasing allele was associated with a ~ 5% increased risk for severe CAD. As T2D is a complex disease determined by multiple variants, the genetic propensity might be estimated more accurately by using an overall GRS approach. Consequently, consistent with previous studies [27, 38], we utilized a GRS to evaluate the overall susceptibility to T2D based on 41 well-established T2D-predisposing variants identified from GWAS. By accumulation, individuals in the upper tertile of GRS had a ~ 40% greater risk of developing severe CAD compared to those in the lower tertile. This significant association provided the opportunity to investigate a causal relationship between T2D and severe CAD using the theory of Mendelian randomization. Since genetic variants are randomly assigned and uncorrelated with environmental factors, the observed genetic associations should be less likely to be affected by confounding factor and reverse causation [39]. As a result, the proxy use of GRS significantly associated with a phenotype instead of the phenotype itself in the association analyses can help to explore a potential causal relationship. Furthermore, the fact that GRS remains significantly associated with the severity of CAD after inclusion of T2D status into the model suggested that the GRS provided additional information than the prevalence of disease in a cross-sectional survey. We hypothesized that the GRS gave more information because it represented the cumulative lifetime exposure to develop T2D. Another explanation was the possible pleiotropic effects of the SNPs in the GRS. Nevertheless, adjusting for BMI, smoking, and status of hypertension and hypercholesterolemia did not attenuate the association between GRS and severe CAD, demonstrating that it is unlikely that these metabolic traits lie on the causal pathway from T2D genetic variants to severity of CAD. Furthermore, to minimize the pleiotropic effect, we further excluded the possible pleiotropic SNPs and found that the association remained significant. However, we could not exclude the possibility that the GRS might be related to other unmeasurable variables in the causal pathway. Consequently, further studies are required to validate our results.

### Results in relation to other studies

Numerous studies have provided several lines in supporting a positive relationship between T2D and CAD risk. Growing observational data has clearly shown that T2D is associated with CAD risk [4–7, 9, 40]. For instance, a collaborative meta-analysis of 102 prospective studies [40] found that T2D was associated with an increased risk of CAD by twofold, and this effect was independent of several conventional risk factors. Furthermore, although large randomized controlled trials (RCTs) assessing the effect of intensive glucose-lowering therapies on the short-term prevention of CAD have conflicting and inconclusive results [41–45], meta-analysis [46] of RCTs suggested a modest reduction in events of CAD. In addition, T2D also showed its significant association with the extent and severity of CAD. Rana et al. [21] demonstrated a significant “step up” increased risk in 3370 diabetic patients compared with 6740 propensity-matched non-DM individuals for one-vessel, two-vessel, and three-vessel disease. Niccoli et al. [47] found that diabetic patients exhibited substantially increased severity of coronary atherosclerosis compared to non-diabetic patients at the time of first ACS. Taken together, in line with our findings, these data support the positive role in T2D on the development and severity of CAD.

Several previous studies concentrated their efforts on 9p21, a well-known risk region for CAD, and revealed its significant association with the severity of CAD [48–50]. However, we could not replicate this association in our data in either rs1333049 or SNPs with high linkage disequilibrium (Additional file 1: Figure S4). The most significant SNP in that region is rs28613732 (*P* = 0.004), a three prime untranslated region (3′-UTR) variant locates at 9:21935808 (hg19). This inconsistency may stem from the diverse ethnicities involved, a difference in the definition of outcomes, and so forth.

### Potential mechanisms

Possible biological mechanism behind this association may be the genetic variants associated with T2D, which may promote coronary atherosclerosis and thus resulted in severe CAD. Recently, Xu et al. [38] performed a Mendelian randomization analysis in 11,385 subjects from a community-based study and demonstrated a significant association between T2D-GRS and brachial-ankle pulse wave velocity, which suggested a plausible causal relationship between T2D and increased arterial stiffness. Moreover, a significant association was also observed between genetic variants of fasting glucose and carotid intima-media thickness in the ARIC study, which revealed the possible causal association between increased fasting glucose and atherosclerosis [51]. Furthermore, a pooled analysis of five intravascular ultrasound studies revealed that patients with T2D tend to have a greater atherosclerotic plaque burden, higher atheroma volume, and smaller coronary artery lumen diameter than those without [52]. Insulin resistance starts to develop before glucose changes, and gradually over time, while hyperglycaemia develops in prediabetes and gets worse with disease progression. States of insulin resistance and hyperglycaemia are involved in multiple processes, including elevating levels of free fatty acids, induction of advanced glycation end products, oxidative stress, mitochondrial dysfunction, and epigenetic modifications which, together, contribute to endothelial dysfunction and inflammation resulting in activation of vascular smooth muscle cells, endothelial cells, and monocytes [53–56].

Previous studies also demonstrated the shared genetic background underlying T2D and CAD. Several genetic variants that confer predisposition to T2D have shown their pleiotropic effect on CAD [57–59]. A bivariate GWAS scan identified 19 loci that were associated with both T2D and CAD, which included many established loci for T2D or CAD (e.g. TCF7L2, MRAS, and HNF1A) [60]. A recent comprehensive and data-driven analysis integrated evidence from different omics layers and identified several pathways and gene networks shared in the pathogenesis of CAD and T2D [61]. This study not only confirmed the importance of several well-established processes, including lipid metabolism and immune response, but also pointed out previously under-appreciated processes such as branched-chain amino acids, the neuronal system, and extracellular matrix for both diseases. Therefore, these lines of evidence support the shared genetic contribution of T2D and CAD, which is likely to be due to various shared pathways and risk factors that exert effects on both of the disorders [62, 63].

### Strengths and limitations

The strengths of our study include comprehensive clinical information collection, high quality of genotype data, and a sophisticated GRS construction strategy that selected common variants with robust association with risk of T2D in East Asian people. However, there are also several limitations in the present study. Firstly, we used the number of coronary arteries with at least 50% diameter stenosis to measure the severity of CAD in our study. We acknowledged that this does not provide sufficient information on the volume or area of atherosclerotic plaque and is not sensitive enough for identification of less severe lesions, compared with other scoring systems (e.g. Gensini score and Duke coronary score). However, the objective of our study was to explore the relationship between T2D-GRS and the extent of advanced coronary lesions. Secondly, several previous studies have confirmed the association between poor blood glucose control and CAD severity [64, 65]. However, HbA1c, a marker assessing glycaemic control in patients with diabetes, was not recorded in our study. This means that we failed to test the effect size of T2D-GRS on the extent of CAD after stratification by glycaemic control condition. In addition, although the GRS captured the combined information of genetic propensity to T2D from the well-established T2D-associated variants so far, it accounts for only a small fraction of T2D variation [12]. Lastly, our study is restricted to Chinese subjects and constructed the GRS using the SNPs that were robustly associated with T2D in East Asians. The association between genotype score of T2D and severity of CAD in other ethnic groups should be generalized with caution and remains to be investigated in future studies.

## Conclusions

In conclusion, we found that the genetic predisposition to T2D was significantly associated with greater severity of coronary atheromatous burden in patients with ACS, independent of traditional risk factors. Our findings support a potential causal relationship between T2D and severity of CAD. Further large-scale, prospective studies are needed to validate our findings.

## Supplementary information

**Additional file 1: Table S1.** Criteria for diagnosis of STEMI, NSTEMI and UA. **Table S2.** Characteristics of 42 T2D SNPs and their associations with T2D (adjusted for age, sex and BMI). **Table S3.** The associations of severity of CAD in relation to unweighted GRS and GRS after excluding SNPs with pleiotropic effects. **Figure S1.** Distribution of the number of T2D risk increasing risk alleles (X-axis) and the percentage of T2D by the GRS. The columns and left Y-axis indicated the number of the participants according to different GRS; the dots and right Y-axis indicated the percentage of the T2D patients according to different GRS. **Figure S2.** Distribution of the number of T2D risk increasing risk alleles (X-axis) and the mean fasting glucose by the GRS. The columns and left Y-axis indicated the number of the participants according to different GRS; the dots and error bars indicated mean (± standard error) fasting glucose according to different GRS. **Figure S3.** Association of each T2D risk SNP and multi-vessel disease. Data were presented as OR and 95% CI. *P* values were calculated from logistic regression model with the severity of CAD as dependent variable and each SNP as independent variable in an additive genetic model after adjustment for age and sex. **Figure S4.** Regional association plots with association between rs1333049 and the severity of CAD. Associations of individual variants are plotted as -log10 P against chromosomal position. The right Y-axis shows the recombination rate estimated from the 1000 Genomes Project CHB and JPT data.

## Data Availability

Data of the current study is available from the corresponding author on reasonable request.

## Abbreviations

ACS: acute coronary syndromes
BMI: body mass index
CAD: coronary artery disease
CI: confidence interval
CVD: cardiovascular disease
GRS: genetic risk score
GWAS: genome-wide association studies
HDL: high-density lipoprotein
HWE: Hardy–Weinberg equilibrium
LD: linkage disequilibrium
LDL: low-density lipoprotein
MAF: minor allele frequency
MI: myocardial infarction
NSTEMI: non-ST-segment elevation myocardial infarction
OR: odds ratio
SD: standard deviation
STEMI: ST-segment elevation myocardial infarction
SNP: single nucleotide polymorphism
T2D-GRS: type 2 diabetes genetic risk score
TC: total cholesterol
TG: triglyceride
UA: unstable angina
WHR: waist-hip ratio
