# Supplementary material for: Genetic predisposition to type 2 diabetes is associated with severity of coronary artery disease in patients with acute coronary syndromes
Source: Cardiovasc Diabetol. 2019 Oct 8;18:131. doi: 10.1186/s12933-019-0930-1 (PMC6784340; doi:10.1186/s12933-019-0930-1)

**Table S1.** Criteria for diagnosis of STEMI, NSTEMI and UA

| **STEMI** | 1. History of chest pain/discomfort *and* 2. Persistent ST-segment elevation (>30 min) of ≥0.1 mV in two or more contiguous ECG leads or presumed new left bundle branch block on admission *and* 3. Elevation of cardiac biomarkers (CK-MB, troponins): at least one value above the 99th percentile of the local laboratory upper reference limit |
| --- | --- |
| **NSTEMI** | 1. History of chest pain/discomfort *and* 2. Lack of persistent ST-segment elevation, left bundle branch block or intraventricular conduction disturbances *and* 3. Elevation of cardiac biomarkers (CK-MB, troponins): at least one value above the 99^th^ percentile of the upper reference limit |
| **UA** | 1. Symptoms of angina at rest or on minimal exercise *and* 2. At least 0.5 mm ST deviation in at least two leads *and* 3. No increase in biomarkers of necrosis *or* 4. Objective evidence of ischemia by non-invasive imaging *or* significant coronary stenosis as determined by the treating physician at angiography if standard practice at the study site |

Abbreviations: CK-MB, creatine kinase muscle and brain; ECG, electrocardiogram; NSTEMI, non-ST-segment elevation myocardial infarction; STEMI, ST-segment elevation myocardial infarction; UA, unstable angina.

**Table S2.** Characteristics of 42 T2D SNPs and their associations with T2D (adjusted for age, sex and BMI).

| Chr. | Position | Locus | SNP | Effect Allele | Reference Allele | EAF | Known OR from literature | *P* for HWE | T2D | | Pleiotropic  effect |
| --- | --- | --- | --- | --- | --- | --- | --- | --- | --- | --- | --- |
|  |  |  |  |  |  |  |  |  | OR (95%CI) | *P* value |  |
| 1 | 214159256 | PROX1 | rs340874 | C | T | 0.350 | 1.080 | 0.533 | 1.05 (0.86-1.27) | 0.633 |  |
| 1 | 120517959 | NOTCH2 | rs10923931 | T | G | 0.040 | 1.110 | 1.000 | 1.27 (0.74-2.20) | 0.387 |  |
| 2 | 165528876 | GRB14 | rs13389219 | C | T | 0.890 | 1.200 | 0.027 | 1.02 (0.75-1.37) | 0.916 | WHR |
| 2 | 60584819 | BCL11 | rs243021 | A | G | 0.670 | 1.050 | 0.757 | 0.92 (0.75-1.13) | 0.418 |  |
| 2 | 227093745 | IRS1 | rs2943641 | C | T | 0.920 | 1.120 | 0.403 | 0.86 (0.59-1.27) | 0.454 |  |
| 2 | 27741237 | GCKR | rs780094 | C | T | 0.420 | 1.050 | 0.775 | 1.19 (0.99-1.44) | 0.067 | TC, LDL |
| 2 | 43732823 | THADA | rs7578597 | T | C | 0.993 | 1.181 | 0.079 | 1.28 (0.48-3.39) | 0.617 |  |
| 3 | 12393125 | PPARG | rs1801282 | C | G | 0.960 | 1.130 | 0.109 | 1.07 (0.74-1.55) | 0.719 | BMI |
| 3 | 185511687 | IGF2BP2 | rs4402960 | T | G | 0.300 | 1.070 | 0.672 | 1.08 (0.87-1.33) | 0.494 |  |
| 3 | 123065778 | ADCY5 | rs11708067 | A | G | 0.993 | 1.490 | 1.000 | 0.22 (0.03-1.66) | 0.141 |  |
| 4 | 1309901 | MAEA | rs6815464 | C | G | 0.580 | 1.130 | 0.066 | 0.83 (0.67-1.02) | 0.072 |  |
| 5 | 55806751 | ANKRD55 | rs459193 | G | A | 0.510 | 1.140 | 0.134 | 0.98 (0.82-1.17) | 0.824 | BMI, WHR, CAD, TC, HDL |
| 6 | 20685486 | CDKAL1 | rs9356744 | C | T | 0.390 | 1.200 | 0.846 | 1.14 (0.95-1.38) | 0.163 | BMI |
| 6 | 39284050 | KCNK16 | rs1535500 | T | G | 0.420 | 1.080 | 0.831 | 1.03 (0.86-1.24) | 0.737 |  |
| 7 | 28180556 | JAZF1 | rs864745 | T | C | 0.800 | 1.060 | 0.778 | 1.01 (0.81-1.24) | 0.949 |  |
| 7 | 15064309 | DGKB | rs2191349 | T | G | 0.720 | 1.110 | 0.820 | 0.92 (0.76-1.12) | 0.426 |  |
| 7 | 127246903 | PAX4 | rs10229583 | G | A | 0.780 | 1.200 | 1.000 | 0.76 (0.57-1.02) | 0.069 |  |
| 8 | 118184783 | SLC30A8 | rs13266634 | C | T | 0.540 | 1.110 | 0.474 | 0.98 (0.81-1.18) | 0.835 | TC |
| 8 | 95960511 | TP53INP1 | rs896854 | T | C | 0.240 | 1.070 | 0.813 | 0.88 (0.72-1.08) | 0.222 |  |
| 8 | 41519462 | ANK1 | rs515071 | G | A | 0.770 | 1.140 | 0.051 | 0.93 (0.70-1.22) | 0.581 |  |
| 9 | 22134094 | CDKN2A/B | rs10811661 | T | C | 0.280 | 1.210 | 0.950 | 0.91 (0.76-1.10) | 0.333 | CAD, BMI |
| 9 | 8879118 | PTPRD | rs17584499 | T | C | 0.090 | 1.570 | 0.076 | 1.14 (0.83-1.57) | 0.432 |  |
| 9 | 4287466 | GLIS3 | rs7041847 | A | G | 0.410 | 1.100 | 0.000 | 1.11 (0.91-1.35) | 0.314 |  |
| 9 | 84308948 | TLE1 | rs2796441 | G | A | 0.430 | 1.080 | 0.443 | 0.98 (0.81-1.19) | 0.873 |  |
| 10 | 114758349 | TCF7L2 | rs7903146 | T | C | 0.020 | 1.160 | 0.178 | 0.64 (0.38-1.07) | 0.088 | BMI |
| 10 | 80942631 | ZMIZ1 | rs12571751 | A | G | 0.520 | 1.160 | 0.957 | 1.03 (0.86-1.25) | 0.722 |  |
| 10 | 94462882 | HHEX/IDE | rs1111875 | C | T | 0.370 | 1.110 | 0.119 | 0.99 (0.79-1.24) | 0.918 |  |
| 10 | 12314997 | CDC123/CAMK1D | rs10906115 | A | G | 0.560 | 1.130 | 0.573 | 1.00 (0.83-1.21) | 0.996 |  |
| 11 | 2839751 | KCNQ1 | rs2237892 | C | T | 0.610 | 1.420 | 0.667 | 0.98 (0.80-1.20) | 0.845 | BMI |
| 11 | 72433098 | ARAP1 | rs1552224 | A | C | 0.930 | 1.160 | 0.458 | 1.08 (0.77-1.53) | 0.646 |  |
| 11 | 17408630 | KCNJ11 | rs5215 | C | T | 0.390 | 1.130 | 0.824 | 1.18 (0.98-1.43) | 0.082 | BMI |
| 12 | 27965150 | KLHDC5 | rs10842994 | C | T | 0.740 | 1.110 | 0.269 | 0.81 (0.63-1.04) | 0.095 |  |
| 13 | 80717156 | SPRY2 | rs1359790 | G | A | 0.720 | 1.150 | 0.392 | 0.85 (0.69-1.05) | 0.131 |  |
| 15 | 62396389 | C2CD4A/C2CD4B | rs7172432 | A | G | 0.550 | 1.120 | 0.036 | 0.92 (0.76-1.10) | 0.365 |  |
| 15 | 77747190 | HMG20A | rs7178572 | G | A | 0.390 | 1.080 | 1.000 | 1.29 (1.06-1.57) | 0.010 |  |
| 15 | 91521337 | PRC1 | rs8042680 | A | C | 0.995 | 1.670 | 1.000 | 0.28 (0.09-0.91) | 0.034 |  |
| 16 | 53813367 | FTO | rs17817449 | G | T | 0.140 | 1.181 | 0.617 | 1.36 (1.03-1.78) | 0.028 | BMI |
| 17 | 36098040 | TCF2(HNF1B) | rs4430796 | G | A | 0.280 | 1.110 | 0.341 | 1.44 (1.19-1.75) | 2.2×10^-4^ |  |
| 17 | 2216258 | SRR | rs391300 | C | T | 0.770 | 1.280 | 0.613 | 0.97 (0.79-1.19) | 0.756 |  |
| 17 | 6945940 | SLC16A11 | rs13342232 | G | A | 0.180 | 1.399 | 0.305 | 1.23 (0.94-1.62) | 0.133 |  |
| 18 | 57884750 | MC4R | rs12970134 | A | G | 0.170 | 1.070 | 0.868 | 1.01 (0.80-1.27) | 0.962 | BMI |
| 19 | 33893008 | PEPD | rs3786897 | A | G | 0.560 | 1.100 | 1.000 | 1.01 (0.82-1.24) | 0.941 | BMI, WHR |

**Table S3.** The associations of severity of CAD in relation to unweighted GRS and GRS after excluding SNPs with pleiotropic effects.

|  | N | Prevalence of multi-vessel disease | OR (95%CI) | *P* value | *P* for trend |
| --- | --- | --- | --- | --- | --- |
| Unweighted GRS |  |  |  |  |  |
| Low risk | 472 | 62.3% | 1 (ref) |  |  |
| Medium risk | 471 | 64.5% | 1.09 (0.83-1.44) |  |  |
| High risk | 471 | 69.2% | 1.34 (1.02-1.78) |  | 0.037 |
| Continuous (Per SD) | 1414 | 65.3% | 1.21 (1.08-1.36) | <0.001 |  |
| GRS _no pleiotropic SNP_ |  |  |  |  |  |
| Low risk | 472 | 60.8% | 1 (ref) |  |  |
| Medium risk | 471 | 66.5% | 1.24 (0.94-1.3) |  |  |
| High risk | 471 | 68.8% | 1.31 (0.99-1.72) |  | 0.047 |
| Continuous (Per SD) | 1414 | 65.3% | 1.14 (1.02-1.28) | 0.021 |  |

Adjust for age, sex, BMI, smoking, hypertension, and hypercholesterolemia.

**Figure S1.** Distribution of the weighted T2D-GRS (X-axis) and the percentage of T2D patients by GRS. The columns and left Y-axis indicated the number of participants according to different GRS; the dots and right Y-axis indicated the percentage of T2D patients according to different GRS.


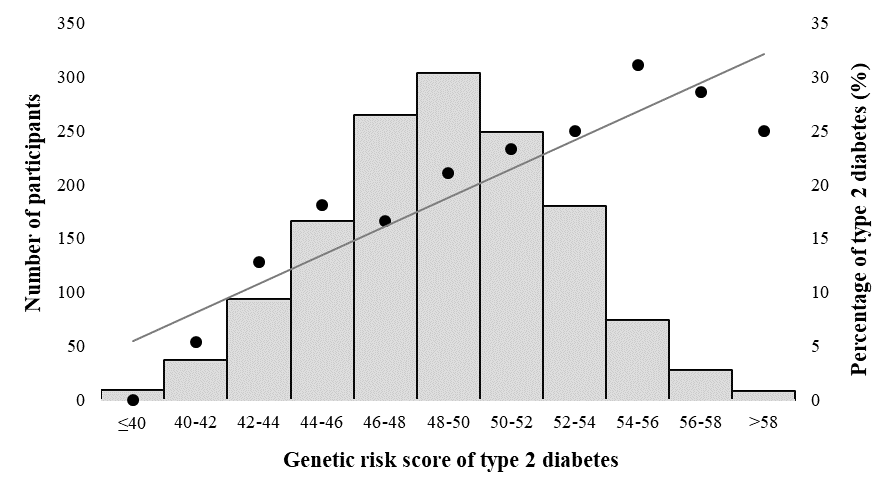


**Figure S2.** Distribution of the weighted T2D-GRS (X-axis) and mean fasting glucose level by GRS in participants without antidiabetic medication usage. The columns and left Y-axis indicated the number of participants according to different GRS; the dots indicated mean (±standard error) of fasting glucose according to different GRS.


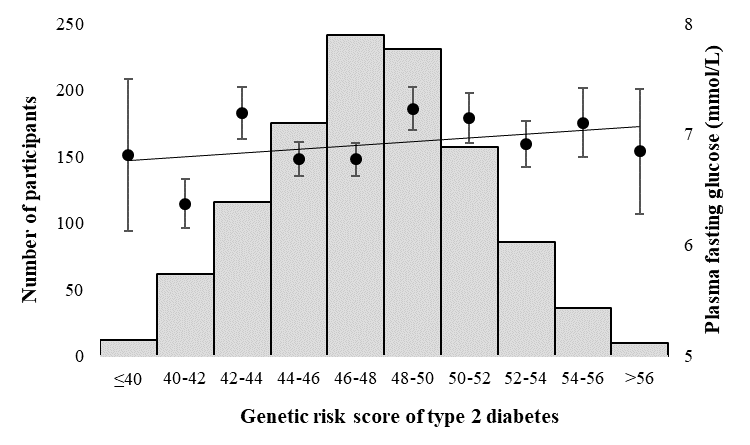


**Figure S3.** Association of each T2D risk SNP and multi-vessel disease. Data were presented as OR and 95% CI. *P* values were calculated from logistic regression model with the severity of CAD as dependent variable and each SNP as independent variable in an additive genetic model after adjustment for age and sex.

**Figure S4.** Regional association plots with association between rs1333049 and the severity of CAD. Associations of individual variants are plotted as -log10 P against chromosomal position. The right Y-axis shows the recombination rate estimated from the 1,000 Genomes Project CHB and JPT data.


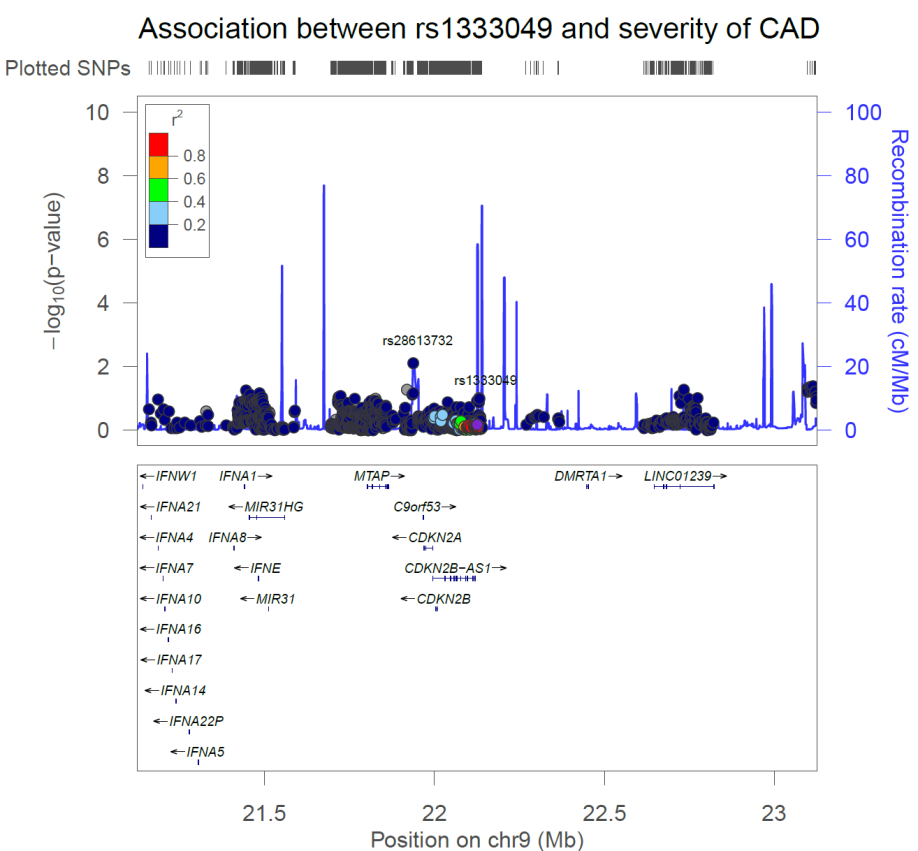

Supplement: Supplementary file 1 — Additional file 1: Table S1. Criteria for diagnosis of STEMI, NSTEMI and UA. Table S2. Characteristics of 42 T2D SNPs and their associations with T2D (adjusted for age, sex and BMI). Table S3. The associations of severity of CAD in relation to unweighted GRS and GRS after excluding SNPs with pleiotropic effects. Figure S1. Distribution of the number of T2D risk increasing risk alleles (X-axis) and the percentage of T2D by the GRS. The columns and left Y-axis indicated the number of the participants according to different GRS; the dots and right Y-axis indicated the percentage of the T2D patients according to different GRS. Figure S2. Distribution of the number of T2D risk increasing risk alleles (X-axis) and the mean fasting glucose by the GRS. The columns and left Y-axis indicated the number of the participants according to different GRS; the dots and error bars indicated mean (± standard error) fasting glucose according to different GRS. Figure S3. Association of each T2D risk SNP and multi-vessel disease. Data were presented as OR and 95% CI. P values were calculated from logistic regression model with the severity of CAD as dependent variable and each SNP as independent variable in an additive genetic model after adjustment for age and sex. Figure S4. Regional association plots with association between rs1333049 and the severity of CAD. Associations of individual variants are plotted as -log10 P against chromosomal position. The right Y-axis shows the recombination rate estimated from the 1000 Genomes Project CHB and JPT data. [file 12933_2019_930_MOESM1_ESM.docx]
